# The ATP-P2X7 Signaling Pathway Participates in the Regulation of Slit1 Expression in Satellite Glial Cells

**DOI:** 10.3389/fncel.2019.00420

**Published:** 2019-09-19

**Authors:** Quanpeng Zhang, Jiuhong Zhao, Jing Shen, Xianfang Zhang, Rui Ren, Zhijian Ma, Yuebin He, Qian Kang, Yanshan Wang, Xu Dong, Jin Sun, Zhuozhou Liu, Xinan Yi

**Affiliations:** ^1^Department of Anatomy, Hainan Medical University, Haikou, China; ^2^Joint Laboratory for Neuroscience, Hainan Medical University, Fourth Military Medical University, Haikou, China; ^3^Department of Ophthalmology, First Affiliated Hospital of Hainan Medical University, Haikou, China; ^4^Infection Control Department, People’s Hospital of Xing’an County, Guilin, China; ^5^Quality Inspection Department, Minghui Industry (Shenzhen) Co., Ltd., Shenzhen, China; ^6^Hainan Provincial Key Laboratory of Carcinogenesis and Intervention, Hainan Medical University, Haikou, China; ^7^Department of Clinical Medicine, Hainan Medical University, Haikou, China

**Keywords:** Slit1, dorsal root ganglia, P2X7R, satellite glial cells, sciatic nerve crush

## Abstract

Slit1 is one of the known signaling factors of the slit family and can promote neurite growth by binding to its receptor, Robo2. Upregulation of Slit1 expression in dorsal root ganglia (DRG) after peripheral nerve injury plays an important role in nerve regeneration. Each sensory neuronal soma in the DRG is encapsulated by several surrounding satellite glial cells (SGCs) to form a neural structural unit. However, the temporal and spatial patterns of Slit1 upregulation in SGCs in DRG and its molecular mechanisms are not well understood. This study examined the spatial and temporal patterns of Slit1 expression in DRG after sciatic nerve crush by immunohistochemistry and western blotting. The effect of neuronal damage signaling on the expression of Slit1 in SGCs was studied *in vivo* by fluorescent gold retrograde tracing and double immunofluorescence staining. The relationship between the expression of Slit1 in SGCs and neuronal somas was also observed by culturing DRG cells and double immunofluorescence labeling. The molecular mechanism of Slit1 was further explored by immunohistochemistry and western blotting after intraperitoneal injection of Bright Blue G (BBG, P2X7R inhibitor). The results showed that after peripheral nerve injury, the expression of Slit1 in the neurons and SGCs of DRG increased. The expression of Slit1 was presented with a time lag in SGCs than in neurons. The expression of Slit1 in SGCs was induced by contact with surrounding neuronal somas. Through injured cell localization, it was found that the expression of Slit1 was stronger in SGCs surrounding injured neurons than in SGCs surrounding non-injured neurons. The expression of vesicular nucleotide transporter (VNUT) in DRG neurons was increased by injury signaling. After the inhibition of P2X7R, the expression of Slit1 in SGCs was downregulated, and the expression of VNUT in DRG neurons was upregulated. These results indicate that the ATP-P2X7R pathway is involved in signal transduction from peripheral nerve injury to SGCs, leading to the upregulation of Slit1 expression.

## Introduction

It is well established that peripheral nerves can be regenerated (Stoll, 1999; Vogelaar et al., 2004; Scheib and Hoke, 2013; Cattin and Lloyd, 2016; Sanna et al., 2017; Mahar and Cavalli, 2018; Tajdaran et al., 2018; Duraikannu et al., 2019). However, the factors affecting neuron generation are very complicated. In addition to endogenous gene regulation, external factors such as nerve regeneration chambers (Cattin and Lloyd, 2016), neurotrophic factors (Duraikannu et al., 2019), cytokines (Fitzgerald and McKelvey, 2016) and inflammation responses (Cattin and Lloyd, 2016) should also be taken into account. Intensive previous studies have focused on Schwann cells (SCs) (Court et al., 2008; Nave and Trapp, 2008; Parrinello et al., 2010; Arthur-Farraj et al., 2012; Fontana et al., 2012; Beirowski et al., 2014; Kang et al., 2014; Painter et al., 2014; Rosenberg et al., 2014; Gomez-Sanchez et al., 2015; Isaacman-Beck et al., 2015; Jessen et al., 2015), while little research has been conducted on satellite glial cells (SGCs), a type of glial cells in the ganglion, to study their involvement in nerve regeneration (Hanani, 2005). SGCs are flattened glial cells in the peripheral nervous system that encircle the neuronal bodies in sensory ganglia, thus supporting and protecting sensory neurons (Hanani, 2005). SGCs are laminar and have no true processes (Hanani, 2005). Each sensory neuronal soma is encapsulated by several SGCs to form a unique SGCs health; thus, the neuron and its surrounding SGCs form a structural unit (Hanani et al., 2002; Hanani, 2005, 2015; Wang et al., 2016). Inside the dorsal root ganglion (DRG), SGCs provide support for neurons by mediating the response against inflammation (Hanani, 2005) and synthesizing multiple neurotrophic factors to support the survival of the neurons (Hanani, 2010, 2012; Hanani et al., 2014). Slit1 is one of the known signaling factors of the slit family and can guide both axon projection and neuronal migration (Blockus and Chedotal, 2016). Slit can guide axon projection by mediating the branching of the sensory axon growth cone (Ma and Tessier-Lavigne, 2007). In a previous study (Yi et al., 2006), we observed that Slit1 expression was increased in the SGCs of damaged DRG. However, SGCs express specific patterns of Slit1, and the signal transduction mechanism between neurons and SGCs was not identified. Signal communication between neurons and SGCs is bidirectional (Christie et al., 2015). The interacting molecules include adenosine triphosphate (ATP) (Zhang et al., 2007), nitric oxide (NO) (Bradman et al., 2010), endothelin 1 (Giaid et al., 1989; Milner and Burnstock, 2000; Hanani, 2012), glutamic acid (Kung et al., 2013) and calcium ions (Suadicani et al., 2010; Castillo et al., 2011). ATP is a neurotransmitter secreted by many cell types including sensory neurons (Chaudhari, 2014), and it participates in signal transduction between neurons and SGCs (Zhang et al., 2007). ATP is stored in many types of neurons and is released not only at synapses but also in axons and cell bodies (Zhang et al., 2007). ATP is an important extracellular signaling molecule that communicates through complex purine energy signaling pathways (Burnstock, 2006). This signaling pathway consists of many membrane receptors and extracellular enzymes including the P2X7 receptor (P2X7R), which belongs to the P2X family (Yegutkin, 2008). P2X7R, an important member of the purine receptor family, is a trimer ATP-gated cation channel encoded by the P2X7R gene (Coddou et al., 2011). P2X7R can be activated by extracellular ATP, and it participates in the regulation of cell biological functions such as cell signaling pathways, cytokine secretion, and the growth and apoptosis of cells (Sluyter, 2017). P2X7R expressed in the SGCs is involved in DRG neuron – SGC communication in adult rats (Chen et al., 2012a). P2X7R is the main receptor in SGCs (Zhang et al., 2007; Kushnir et al., 2011; Song et al., 2018). Similar to the closely related dorsal root ganglion neurons, the membrane capacitance of cultured trigeminal ganglion neurons increased significantly after electrical stimulation, which resulted in the release of vesicles content in the extracellular space of the ganglion (Sforna et al., 2019). The increase of membrane capacitance is an indicator of somatic exocytosis. Sforna et al. (2019) identified Ca^2+^ -dependent and Ca^2+^ -independent somatic vesicular release from trigeminal neurons and the Ca^2+^ channel types involved in the process. The vesicular nucleotide transporter (VNUT) is a key molecule for the vesicular storage and nucleotide release of ATP from neutrophils (Sawada et al., 2008). Injured DRG neurons increase the secretion of ATP and VNUT (Goto et al., 2017). After the cell is stimulated, ATP is secreted from the cell body to become an extracellular signaling molecule that binds to purinoreceptors at the surface of SGCs. It triggers intracellular signal transduction (Zhang et al., 2007). ATP is used as the signaling molecule for signal transmission between neurons and SGCs, and SGCs receive signaling stimuli, leading to the production of a series of reactions (Zhang et al., 2007). The molecular mechanisms of the protective effect of SGCs on DRG neurons and the promotion of the regeneration of DRG neurons have not been clarified. In this study, we further clarified the effect of damage signals on the expression of Slit1 in DRG neurons and their SGCs and clarified the role of ATP and its receptors.

## Results

### Expression of Slit1 in Intact DRG Neurons and SGCs *in vivo* and *in vitro*

Preliminary analyses were performed to verify the specificity of the Slit1, microtubule-associated protein 2 (MAP2), glutamine synthetase (GS), activating transcription factor 3 (ATF3) and VNUT and P2X7R antibodies used in this study (Supplementary Figure S1). The expression of MAP2 and Slit1 co-localized in DRG cells subjected to double labeling of Slit1 and MAP2 [neuron biomarker (Pellegrino et al., 2011)], (Supplementary Figures S2A–C). Expression of Slit1 was not observed in the SGCs of intact DRG double labeled for Slit1 and GS [biomarker for SGCs (Donegan et al., 2013)], (Supplementary Figures S2D–F). To observe the expression of Slit1 in cultured neurons and SGCs, double immunofluorescence labeling of Slit1 and MAP2 or GS in cultured DRG cells was performed. The results showed that Slit1 was expressed in the cultured sensory neuronal soma of DRG but not in neurites and weakly in SGCs (Supplementary Figures S2G–L).

### Neural Trunk Injury Can Up-Regulate the Expression of Slit1 in DRG Neurons and SGCs

**FIGURE 1:**
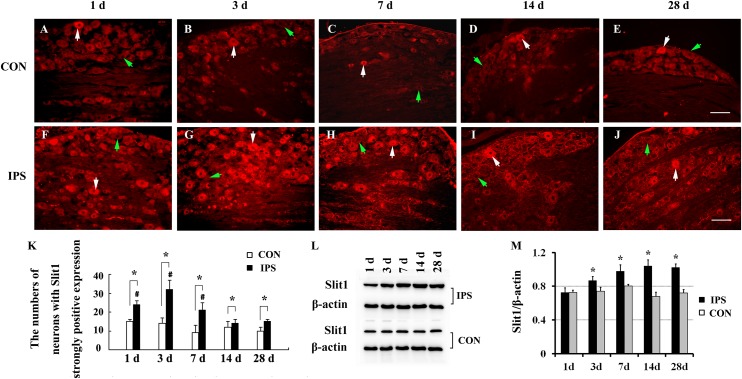
Dynamic changes in Slit1 expression in DRG after sciatic nerve injury. The expression of Slit1 in DRG was detected by immunofluorescence histochemistry and western blotting at different time points after SNC. **(A–E)** Contralateral DRG sections. **(F–J)** Ipsilateral DRG sections. The white arrows indicate strongly Slit1-positive neurons, and the green arrow indicates weakly Slit1-positive neurons. Scale bar = 100 μm. **(K)** At different time points, the numbers of strongly Slit1-positive DRG neurons on both sides were compared, and one-way repeated measures ANOVA and paired *t*-tests were used for statistical analysis. “^∗^” in comparison with the control side, the difference is statistically significant at *P* < 0.05; “#” in comparisons of the groups at 1, 3 or 7 days after injury with the group at 14 days after injury, the difference is statistically at *P* < 0.01. **(L)** Detection of DRG Slit1 protein expression at different time points by western blotting (*N* = 3). The level of β-actin was detected as loading control. **(M)** At different time points, the relative gray values of DRG Slit1 expression on the two sides were compared. *N* = 3, and one-way repeated measures ANOVA and paired *t*-tests were used for statistical analysis, “^∗^” compared to the contralateral side, *P* < 0.01. CON: contralateral DRG, IPS: ipsilateral (crush side) DRG.

**FIGURE 2:**
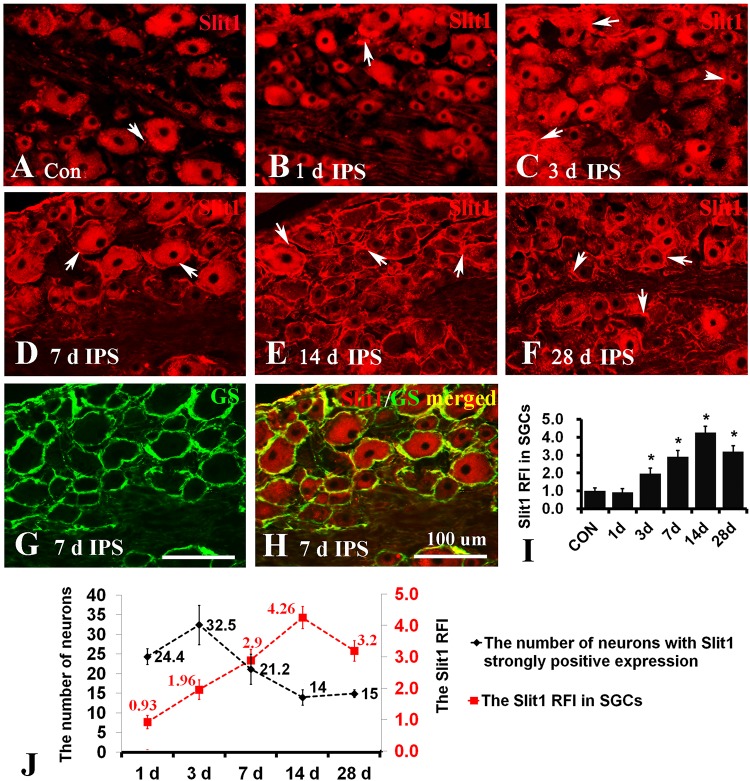
Slit1 expression in SGCs of injured DRG. Slit1 expression in the SGCs of injured DRG was detected by immunofluorescence histochemistry. **(A)** Slit1 (red) immunofluorescence staining of contralateral DRG sections randomly selected from the different groups. **(B–F)** Slit1 immunofluorescence staining of ipsilateral DRG at different time points after operation. The arrow indicates Slit1-positive SGCs. **(G)** GS (green) immunofluorescence staining of DRG sections on the 7th day after sciatic nerve crush. **(H)** Merged images from panels **(D,E)**. **(I)** Summary histograms depicting the changes in the Slit1 RFI in SGCs (one-way repeated measures ANOVA, ^∗^ *P* < 0.01); RFI, relative fluorescence intensity. **(J)** Changes in the number of strongly Slit1-positive DRG neurons and the RFI of Slit1 expression in SGCs at different time points after injury are shown. Scale bar = 100 μm.

To observe the effect of nerve injury on the expression of Slit1 in DRG neurons and SGCs, we used a unilateral rat sciatic nerve crush model (SNC). The immunohistochemistry results revealed that Slit1 was expressed in the sensory neurons on the DRG of both sides. However, the expression of Slit1 in the SNC DRG was higher than that in the contralateral DRG (Figures 1A–J). At days 1, 3, and 7 after SNC, the numbers of strongly Slit1-positive neurons were 24.4 ± 2, 32.5 ± 5, and 21.2 ± 3.8, respectively, and a significant difference was observed compared with the contralateral side (*P* < 0.05, Figure 1K). The number of Slit1-positive neurons was increased at day 1 and peaked at day 3, followed by a gradual decrease and a return to normal levels at day 14 after SNC (Figure 1K). The results of Slit1 western blotting after SNC (Figures 1K,L) were basically consistent with the immunohistochemistry results (Figure 1L). Slit1 expression was not observed in SGCs in intact DRG (Figure 2A) and was observed occasionally in SGCs at day 1, then increased between day 3 and day 14 after SNC according to double immunofluorescence labeling of Slit1 and GS (Figures 2C–F and Supplementary Figure S4). Although the level of Slit1 expression was decreased at day 28 after SNC, it was still higher than in the contralateral DRG (Figures 2C–F). At each time point after injury, the Slit1 relative fluorescence intensity (RFI) in SGCs on the ipsilateral side was higher than that on the opposite side, and the difference was statistically significant according to one-way ANOVA (*P* < 0.001) (Figure 2I). Interestingly, following SNC, the peak expression of Slit1 in SGCs appeared later than in the neuronal somas (Figure 2J).

### A Neuronal Damage Signal Can Induce Up-Regulation of Slit1 Expression in SGCs in the Structural Unit

**FIGURE 3:**
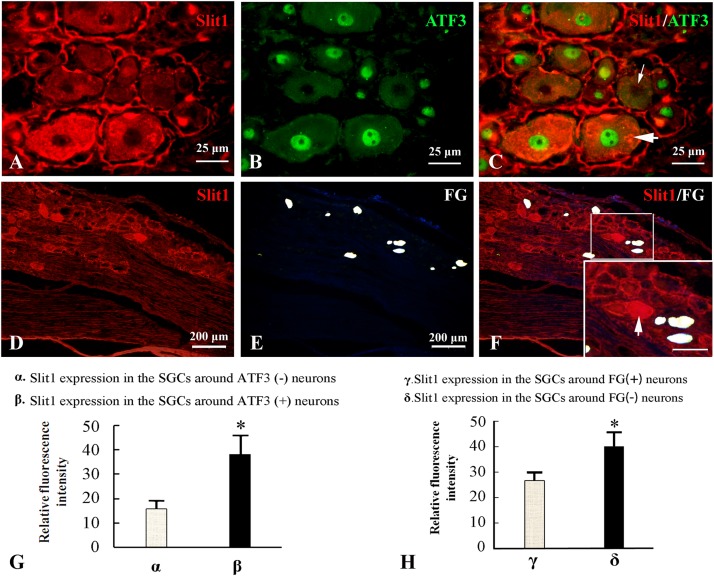
Effects of DRG-injured neuronal soma on Slit1 expression in SGCs. The Slit1 and ATF3 double immunofluorescence labeling method and the Slit1 and FG retrograde labeling method were used to show the expression of Slit1 in SGCs around normal and injured neurons. **(A–C)** Double immunofluorescence labeling of Slit1 and ATF3 was carried out on DRG sections from 7 days after sciatic nerve crush. **(A)** Slit1 (red) immunofluorescence staining. **(B)** ATF3 (green) immunofluorescence staining. **(C)** Merged images of panels **(A,B)**. The thick arrow indicates ATF3-positive neurons, and the thin arrow indicates ATF3-negative neurons, scale bar = 25 μm. **(D–F)** Slit1 immunofluorescence staining and FG retrograde labeling. **(D)** DRG Slit1 immunofluorescence on the 7th day after the operation. **(E)** FG (white)-traced positive neurons. **(F)** Merged images of panels **(D,E)**. Scale bar = 200 μm. The large framed image is an enlargement of the small frame, and thin arrows indicate FG-negative neurons in panels **(F)**. **(G)** Summary histograms depict the changes in the RFI of Slit1 in SGCs surrounding ATF3-positive and negative neurons determined with paired *t*-tests, ^∗^ *P* < 0.001; bars represent the standard error of the mean; RFI, relative fluorescence intensity. **(H)** Summary histograms depict the changes in the RFI of Slit1 expression in SGCs surrounding FG-negative and FG-positive neurons determined with paired *t*-tests, ^∗^*P* < 0.001; bars represent the standard error of the mean; RFI, relative fluorescence intensity.

Because the nerve crush model only causes partial damage to DRG neurons, it is convenient to observe the effect of neuron damage signals on SGCs in the anatomical unit of neurons. Fluor-Gold (FG) is a slow retrograde axonal tracer, a volume of 0.1 μl of 4% FG solution was inserted into the epineurium 2 mm from the distal to the injured site of the sciatic nerve and the neurons that are reached by retrograde FG transport can be considered uninjured neurons. Additionally, ATF3 was used as an immunofluorescence marker for injured neuronal somas (Donegan et al., 2013). The combination of Slit1 immunofluorescence and the FG retrograde labeling method with Slit1 and ATF3 double immunofluorescence labeling was used to show the expression of Slit1 in SGCs around normal or injured neurons. 7 days after SNC, L5-6 DRG from the injured side were subjected to Slit1 immunofluorescence staining. The results showed that the expression of Slit1 in SGCs around ATF3-positive neurons was significantly higher than that around ATF3-negative neurons (Figures 3A–C). Conversely, the expression of Slit1 in SGCs around FG-positive neurons was significantly lower than that around FG-negative neurons (Figures 3D–F). According to analysis of the RFI with the paired *t*-test at a significance level of *P* < 0.01 (Figures 3G,H).

### Contact of Neuronal Soma With SGCs Induces the Expression of Slit1

**FIGURE 4:**
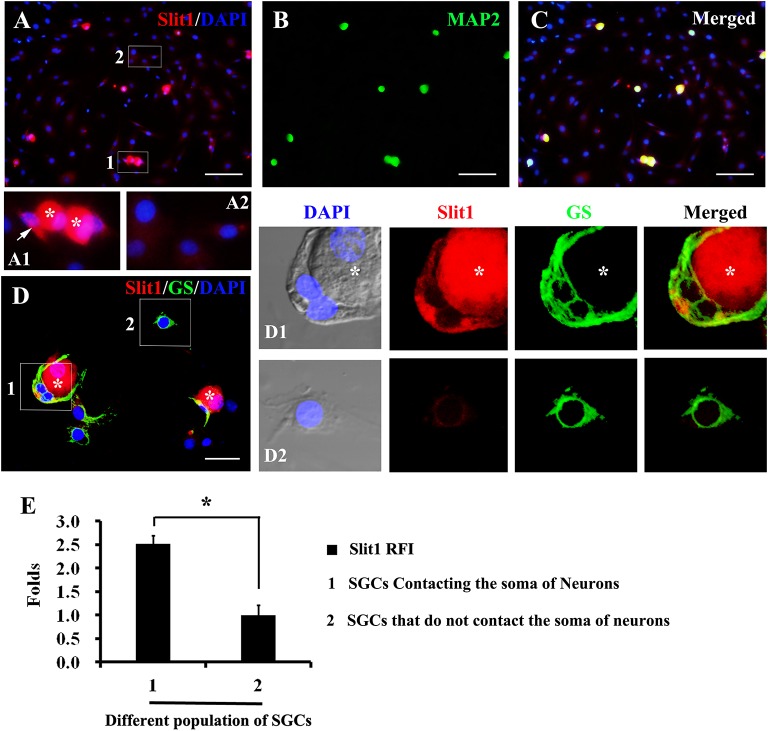
Expression of Slit1 in SGCs directly in contact with the neuronal soma *in vitro* **(A–C)** Primary cultured DRG cells were double labeled with Slit1 and MAP2 immunochemistry, nuclear staining by DAPI. **(A)** Slit1 (red) and DAPI (blue) merged image. The box 1 shows SGCs in contact with neuron bodies, Panel **(A1)** is its enlarged image, with arrows indicating SGCs in contact with neuronal soma (white asterisks); and the box 2 shows SGCs located far from neuron bodies as showed in its enlarged image **(A2)**. **(B)** MAP2 (green) image. **(C)** Slit1 (red), MAP2 (green) and DAPI (blue) merged image. **(D)** Slit1 and GS double immunostaining were performed on cultured DRG cells, nuclear staining by DAPI. The box 1 shows SGCs in contact with Slit1-positive neuronal soma (white asterisks). The box 2 shows SGCs located far from neuron bodies. Panels **(D1,D2)** lines of pictures were local enlarged images of the box 1 and box 2 showed in panel **(D)**, respectively. The white asterisk indicates the neuronal soma. Slit1 (red), MAP2 (green) and DAPI (blue) were showed in panels **(D,D1,D2)**. **(E)** Summary histograms depict the changes in Slit1 expression in two different populations of SGCs, ^∗^ shows the expression of Slit1 in SGCs of population 1 compared with that in SGCs of population 2 analyzed with paired *t*-tests, ^∗^ *P* < 0.001; bars represent the standard error of the mean; RFI, relative fluorescence intensity. Scale bar = 50 μm was showed in panels **(A–C)**; Scale bar = 15 μm was showed in panel **(D)**.

In the process of DRG cell culture, an interesting phenomenon was found. When cultured for 2–7 h, some glial cells detached from the neuronal soma, while others remained in contact with the neuronal soma (Figures 4A,D). Significantly, glial cells that maintained contact with neuronal somas exhibited a strong expression of Slit1, while those located far from neurons presented very weak expression of Slit1 (Figures 4A,D). Immunofluorescence showed that both populations of glial cells expressed GS. RFI analysis showed that the expression of Slit1 in SGCs in contact with neurons was stronger than that in SGCs located far from neurons (Figure 4E) (2.51 ± 0.19 folds, *P* < 0.01).

### Damage Signaling Induces an Increase in VNUT in DRG Neurons

**FIGURE 5:**
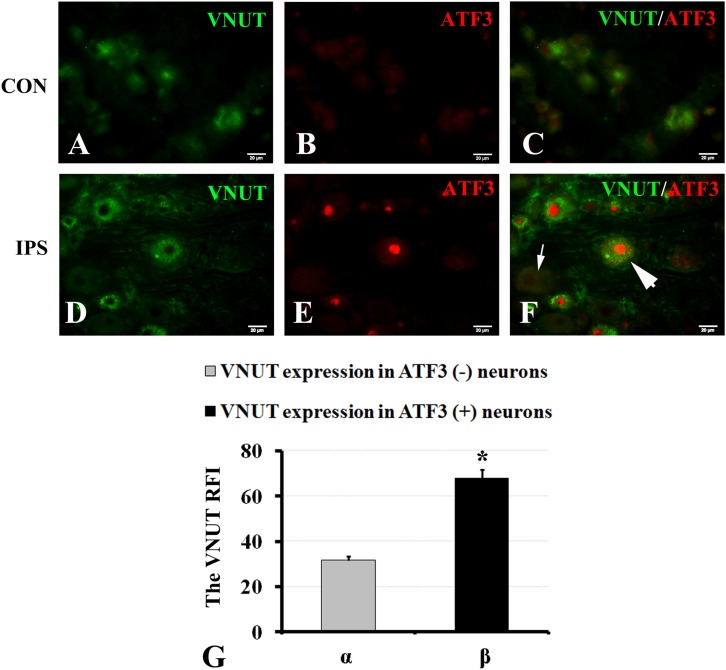
Upregulation of VNUT expression in injured neurons. At 7 days after right sciatic nerve crush, double labeling of VNUT and ATF3 was performed in DRG sections. **(A–C)** Contralateral DRG images, **(D–F)** ipsilateral DRG images following sciatic nerve crush. Panels **(B,E)** immunofluorescence staining for ATF3 (red); Panels **(C,F)** merged images of panels **(A,B)** or **(D,E)**, respectively. A thick arrow indicates ATF3-positive neurons, and a thin arrow indicates ATF3-negative neurons; scale bar = 20 μm. **(G)** Summary histograms depicting the changes in VNUT expression in ATF3-positive neurons and ATF3-negative neurons on the crush side with the paired *t*-test, α and β, ^∗^ *P* < 0.001; bars represent the standard error of the mean; RFI, relative fluorescence intensity.

It has been reported that the changes in the contents of VNUT and ATP are positively associated (Yuri et al., 2013; Menéndez-Méndez et al., 2015; Pérez de Lara et al., 2015; Harada et al., 2018). To observe the effect of an injury signal on ATP production in DRG neurons, we chose to evaluate the changes in VNUT. Double-labeling of ATF3 and VNUT showed that both were co-expressed after SNC (Figures 5A–G). Compared to the RFI of VNUT in ATF3-negative neurons, VNUT expression in ATF3-positive neurons was stronger than that in ATF3-negative neurons (Figure 5H) (*P* < 0.01).

### After Inhibiting the P2X7 Receptor, the Expression of VNUT Increased, While the Expression of Slit1 in SGCs Decreased

**FIGURE 6:**
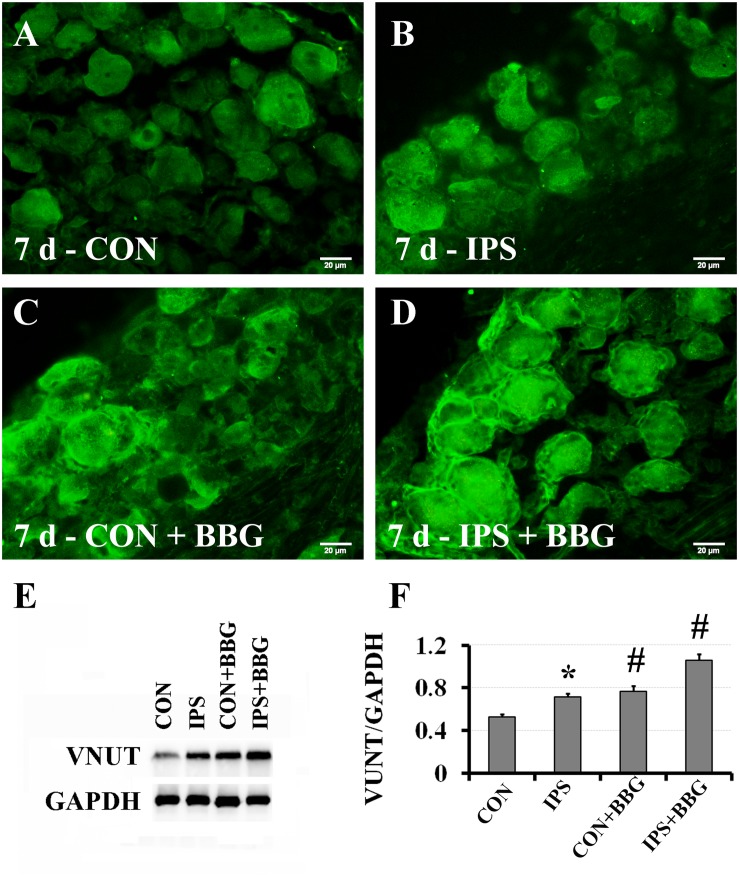
Upregulation of VNUT expression after inhibition of the P2X7 receptor with BBG. After right sciatic nerve crush, BBG (P2X7 receptor inhibitor) was injected intraperitoneally. VNUT immunofluorescence in DRG was detected on the 7th day. **(A–D)** VNUT immunofluorescence staining (green), scale bar = 20 μm. **(E)** The level of VNUT protein was detected by western blotting (*N* = 3). The level of GAPDH was detected as loading control. **(F)** The expression of VNUT before and after BBG treatment on the 7th day after SNC was analyzed with the paired *t*-test, “^∗^” compared with the control side, *P* < 0.001; “#” compared with the contralateral side and the ipsilateral side before BBG treatment, *P* < 0.001. CON: contralateral DRG, IPS: ipsilateral DRG.

**FIGURE 7:**
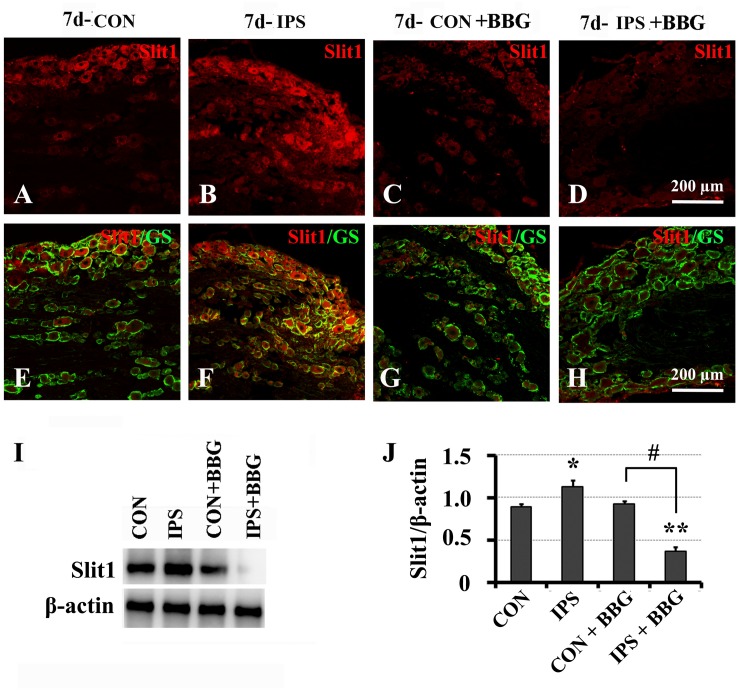
Slit1 expression in SGCs decreased after inhibition of the P2X7 receptor with BBG. After right sciatic nerve crush, BBG (P2X7 receptor inhibitor) was injected intraperitoneally. The expression of Slit1 on the 7th day DRG was detected by double immunofluorescence staining and western blotting. **(A–D)** Slit1 immunofluorescence staining (red). **(E–H)** Merged images of Slit1 (red) and GS (green) immunofluorescence staining. Scale bar = 200 μm. **(I)** the level of Slit1 protein was detected by western blotting (*N* = 3), the level of β-actin was detected as loading control. **(J)** The level of Slit1 before and after BBG treatment on the 7th day post-SNC DRG was analyzed with the paired *t*-test. “^∗^” compared with the control side, the level of Slit1 in IPS side was upregulated, *P* < 0.001; “#” compared with the control side and the ipsilateral side before BBG treatment respectively, the level of Slit1 in “IPS + BBG” was downregulated, *P* < 0.001; The change was not significant in “CON + BBG”, *P* > 0.05. “^∗∗^” compared with “CON + BBG” group, the level of Slit1 in “IPS + BBG” was much lower than that in “CON + BBG” group, *P* < 0.001. CON: contralateral DRG, IPS: ipsilateral DRG.

It has been reported that ATP can interact with the P2X7 receptor on SGC membranes, thus activating a series of molecular events in SGCs (Zhang et al., 2005, 2007; Ryu and McLarnon, 2008; Chen et al., 2012a; Song et al., 2018). BBG is a P2X7 receptor inhibitor (Jiang et al., 2000; Remy et al., 2008; Ryu and McLarnon, 2008). BBG was injected intraperitoneally after unilateral SNC in rats. VNUT immunofluorescence and western blotting showed that the expression of VNUT in DRG neurons on the ipsilateral side increased significantly (Figure 6). After BBG injection, the expression of VNUT in the neurons of both sides was significantly up-regulated (*P* < 0.01) (Figure 6F). Double immunofluorescence labeling was performed by using Slit1 and GS antibodies. The results showed that the expression of Slit1 in DRG neurons and SGCs was increased at 7 days after SNC (Figures 7B,F). After BBG treatment, the expression of Slit1 in neurons and SGCs was downregulated (Figures 7D,H). The results of western blotting detection were similar to those of immunofluorescence (Figure 7I). After BBG injection, the expression of Slit1 on the operated side was significantly lower than that on the contralateral side (Figure 7J) according to a paired *t*-test (*p* < 0.01). Double immunofluorescence labeling was performed with the P2X7R and GS antibodies, and the results showed that the P2X7R and GS antibodies were co-expressed in the SGCs of the DRG of both sides. After BBG injection, no expression of P2X7R was found in DRG on the operated side (Supplementary Figure S3).

## Discussion

This study shows that after peripheral nerve injury, the expression of Slit1 in the neurons and SGCs of DRG increased. The expression of Slit1 was presented with a time lag in SGCs than in neurons. The expression in SGCs of Slit1 was induced by contacting neuronal somas. Through injured cell localization, it was found that the expression of Slit1 was stronger in SGCs surrounding injured neurons than in SGCs surrounding healthy neurons. This result indicates that the damage signal could induce upregulation of Slit1 expression in the SGCs of the DRG neural unit. The expression of VNUT in DRG neurons was increased by the injury signal. After the inhibition of P2X7R, the expression of Slit1 in SGCs was downregulated, and the expression of VNU in DRG neurons was upregulated. These results indicate that the ATP-P2X7R pathway is involved in signal transduction from peripheral nerve injury to SGCs, leading to the upregulation of Slit1 expression. These findings suggest that maintaining the integrity of DRG neurons is beneficial to nerve regeneration and that the ATP-P2X7 pathway between neurons and SGCs plays an important role in the regeneration of peripheral nerves following injury.

Slit, which is generated in glial cells during the development phase, is the guidance molecule for the growth of neurites. There are 4 subtypes of the Slit family: Slit1, Slit2, Slit3 and Slit 4. Slit1, Slit2 and Slit3 all bind to the Robo receptor, which is present in the neuronal cell membrane, to participate in the guidance of axons (Ma and Tessier-Lavigne, 2007; Blockus and Chedotal, 2016; Carr et al., 2017). Previous studies on Slit focusing on SCs suggested that peripheral nerve injury could lead to the expression of various neurotrophic factors in SCs, including NGF, BDNF, neurotrophin 4/5, insulin growth factor 1 and 2 (Scheib and Hoke, 2013), and Slit2 and Slit3 (Carr et al., 2017). Temporarily increased expression of these neurotrophic factors can promote the regeneration of sensory neurons (Scheib and Hoke, 2013). However, we used a mouse polyclonal primary antibody against Slit1 (ab115892, immunogen: Synthetic peptide corresponding to mouse Slit1 aa 497–504 conjugated to keyhole limpet hemocyanin), which was predicted to be able to react with rat Slit1. The results from three repeated experiments were consistent (Supplementary Figures S1, S2), indicating the reliability of the Slit1 primary antibody (ab115892). Through double labeling of Slit1 and MAP2 (neuron biomarker) and Slit1 and GS (biomarker for SGCs), it was found that Slit1 was strongly expressed in the DRG neuronal soma *in vivo* and *in vitro*, but not expressed in SGCs *in vivo* and weakly *in vitro* (Supplementary Figure S2). These results are consistent with the literature (Carr et al., 2017).

In this study, an improved SNC model (Gordon and Borschel, 2017) was used to achieve partial DRG neuron injury, allowing effective evaluation of the regeneration and repair of DRG after neuron injury (Wood et al., 2011; Faroni et al., 2015; Cattin and Lloyd, 2016; Gordon and Borschel, 2017; Mahar and Cavalli, 2018). This model resulted in successful injury of some neurons in the DRG and retention of the integrity of others, which is helpful for observing and comparing Slit1 expression in SGCs located in damaged and undamaged neuron units. In the rat SNC model, DRG neurons are partially injured, that is to say, there are two types of neuron subpopulations in DRG: injured neurons (ATF3 positive, which is a marker of injured neurons) and uninjured neurons (ATF3 negative) (Figure 3 and Supplementary Figure S5). Combining the fluorescence double-label staining data of Slit1 and ATF3, VNUT and ATF3 in this study, we found that there were two mainly types of neuron subpopulations in 7 days post-SNC DRG with using the analytical method of Luigi Catacuzzeno (Catacuzzeno et al., 2014) (Supplementary Table S1). There must be existence of the subpopulations Slit1^–^VNUT^+^ATF3^+^ on injured neurons, it is suggested that VNUT is mainly expressed in injured neurons. Other subpopulations such as Slit1^+^VNUT^+^ATF3^+^, Slit1^+^VNUT^–^ATF3^+^ and Slit1^–^VNUT^–^ATF3^+^ on injured neurons may also exist. Among the uninjured neurons (ATF3 negative), there is only one subpopulation Slit1^–^VNUT^–^ATF3^–^, and no other subpopulations exists. Slit1 or VNUT expression was observed in the uninjured neurons on the control side, which indicated that their expression was not related to the injury signal. It was reported from another study (Seijffers et al., 2006; Carriel et al., 2017) that only a few hours after peripheral nerve injury, neurons exhibit changes in gene and protein expression. Most of the affected genes are associated with neuronal growth factors such as GAP43 and ATF3. ATF3 is used as a marker of injured neurons, and the uninjured neurons do not express ATF3 (Donegan et al., 2013). FG is a slow retrograde axonal tracer with a retrograde tracing speed of approximately 1–12 mm/day. On average, the distance between the sites of SNC and DRG is 35 ± 3 mm in adult rats. Therefore, it was estimated that it would take at least 3 days to reach the DRG, which was in agreement with our experimental results (Figure 3). In this study, FG was injected into the distal point of the injured site to label uninjured neuronal somas through retrograde tracing. ATF3 was used as an immunofluorescence marker for injured neuronal somas. Thus, we could compare the difference in Slit1 expression in the SGCs between the injured neuron units and the undamaged neuron units. We found that the expression of Slit1 was higher in the SGCs that were in contact with injured DRG neuronal somas than in those in contact with uninjured DRG neurons. Many studies (Sebert and Shooter, 1993; Kim et al., 2011) simply include contralateral comparisons, which would not meet our experimental requirements. The experimental animal model and the markers of damaged and undamaged neurons that we chose ensure that we could achieve the goal of the experiment.

The expression of Slit1 in SGCs lags behind that in neurons (Figures 1, 2). One possible explanation is that there is no direct link between DRG glial cells and the peripheral nerve trunk (Arkhipova et al., 2010). The peripheral nerve injury signal must reach the neuronal soma first and is subsequently transmitted to SGCs. In respond to nerve injury, SGCs are activated, and upregulate GFAP protein expression, undergo cell division, activated SGCs generate and release inflammatory cytokines and neurotransmitters such as bradykinin, interleukin-1β (IL-1β), tumor necrosis factor-α (TNF-α), neurotrophins, and ATP into its surroundings (Elson et al., 2004; Zhang et al., 2009). Wu et al. (2017) reported that SGCs were activated and NGF was up-regulated after sciatic nerve injury, the increase was significant on day 7 and 14. This also suggests that it takes a time for SGCs to receive damage signals to be activated and express GFAP and NGF. Therefore, this signal transmission process delays the effect of the injury signal on SGCs. The continuous increase in Slit1 in SGCs coincides with the regeneration of damaged sensory neurons induced by nerve crush injury (Kovacic et al., 2004). Our previous research (Zhang et al., 2010; Chen et al., 2012b) found that Slit1 promotes neuronal neurite outgrowth by binding with Robo2, Slit1- Robo2-srGAP3 pathway plays an important regulatory role in DRG neuron regeneration. In this study, we observed that the expression of Slit1 in SGCs was up-regulated after neuronal injury, which may promote the outgrowth of neuronal processes.

Neurons within the DRG are pseudounipolar neurons, and each sensory neuronal soma is encapsulated by several SGCs to form a unique anatomic unit (Hanani, 2005). Therefore, Hanani (2005) proposed that the unique structure and relationship of SGCs and neuronal somas might have special physiological and pharmacological effects. Thus, we hypothesized that the continuous increase in Slit1 expression observed in the SGCs of DRG is closely associated with this unique anatomical structure. Our experiments showed that 7 days after SNC, Slit1 expression in SGCs was significantly lower in the FG-positive neuron unit than in the FG-negative neuron unit (in which the axon was injured, and axoplasmic transport was blocked); in comparison, Slit1 expression in SGCs was significantly stronger in the ATF3-positive neuron unit than in the ATF3-negative neuron unit (ATF3-negative neuronal somas may indicate uninjured neurons) (Figure 3). This result indicated that upon neuronal injury, Slit1 expression is increased not only in the injured neurons but also in the SGCs of the unit.

Using cultured primary DRG neurons and glial cells and applying double immunofluorescence labeling of Slit1 and GS, we found that the expression of Slit1 in SGCs that accumulated around the neuronal soma was much stronger than that in SGCs located at distal sites in the neuronal somas (Figure 4). This result suggests that direct contact between neuronal somas and SGCs could induce the upregulation of SGC Slit1 expression (Figure 4). SGCs express a series of receptors which can respond to the neurotransmitters released by neurons (Elson et al., 2004; Zhang et al., 2009). Therefore, in the process of cell culture, SGCs contact with neuronal soma, making SGCs more easily activated by signal molecules released by neuron, while SGCs that are not in contact with neuron bodies are less likely to be activated, possibly because the signal molecules are diluted by the culture medium. It was shown that signal transmission between the neuronal soma and SGCs depends on the physical distance, which indirectly indicates that it is very important to maintain the functional relationship between neurons and SGC anatomical units *in vivo*. These results suggest that the transmission of injury signals from the neuronal soma to SGCs is closely associated with the anatomic unit. The molecular mechanisms underlying the transmission of neuronal injury signals to SGCs, which subsequently show increased Slit1 expression, remain unknown.

Signal transmission between neurons and SGCs is bidirectional (Zhang et al., 2007; Kung et al., 2013; Christie et al., 2015). The interacting molecules between neurons and SGCs identified to date include ATP (Zhang et al., 2007), NO (Bradman et al., 2010), glutamic acid (Kung et al., 2013) and calcium ions (Suadicani et al., 2010; Castillo et al., 2011). Additionally, Christie et al. (2015) found that SGCs can sense the injury signal from the distal axons of adjacent neurons via cell enlargement and cell proliferation, and neurons can also transmit some small molecules to the surrounding SGCs. Under pathological and physiological conditions, activation of the receptors of glial cells can promote the interaction between neurons and glial cells (Karadottir et al., 2005; Salter and Fern, 2005; Micu et al., 2006; Castillo et al., 2011). Zhang et al. (2007) reported that ATP released from the neuronal soma activates P2X7 receptors in perineuronal SGCs and triggers communication between neuronal somas and glial cells. They further showed that activation of P2X7 receptors can lead to the release of tumor necrosis factor-α (TNF-α) from SGCs. TNF-α in turn potentiates P2X3 receptor-mediated responses and increases the excitability of DRG neurons. Therefore, we hypothesized that the transmission of damage signals to SGCs may have been achieved through the ATP-P2X7R pathway. The VNUT, which is stored in neurons and releases ATP, was selected as the experimental indicator of changes in the P2X7R pathway. We found that a damage signal could increase the expression of VNUT in DRG neurons. The double immunofluorescence staining of VNUT and ATF3 showed that the expression of VNUT in injured neurons was stronger than that in uninjured neurons (Figure 5, *P* < 0.01). These results indirectly suggest that injured neurons release more ATP than uninjured neurons.

Brilliant blue G (BBG) has been reported to be a P2X7-selective antagonist (Jiang et al., 2000); BBG also shows a neuroprotective effect and no toxicity (Gourine et al., 2005). Furthermore, BBG can be used for intraperitoneal injection *in vivo*, which is simple and convenient (Ryu and McLarnon, 2008; Coddou et al., 2011). Based on these advantages, intraperitoneal injection of BBG, a specific inhibitor of P2X7R, was used as an intervention method. GS and P2X7R double immunofluorescence labeling showed that P2X7R was expressed in SGCs, but P2X7R was not expressed in SGCs after BBG injection (Supplementary Figure S3). Brilliant blue G produced a non-competitive inhibition of rat P2X7 receptors with IC50 values of 10 nM and it exerts a specific inhibitory effect on P2X7 receptor by allosteric modulation (Jiang et al., 2000). The specific antibody of P2X7 receptor could not recognize and bind to the allosteric P2X7 receptor, so the expression of P2X7 receptor could not be detected by immunofluorescence staining after BBG injection. This result illustrates that the peritoneal injection of BBG was effective, and after inhibition of P2X7R, the expression of Slit1 in SGCs was downregulated (Figure 7), and the expression of VNUT in DRG neurons was upregulated (Figure 6). This result may be explained by the increased feedback secretion of the ATP ligand after the receptor is inhibited. It is necessary to further validate this feedback regulation phenomenon using the P2X7 gene knockout method.

## Conclusion

**FIGURE 8:**
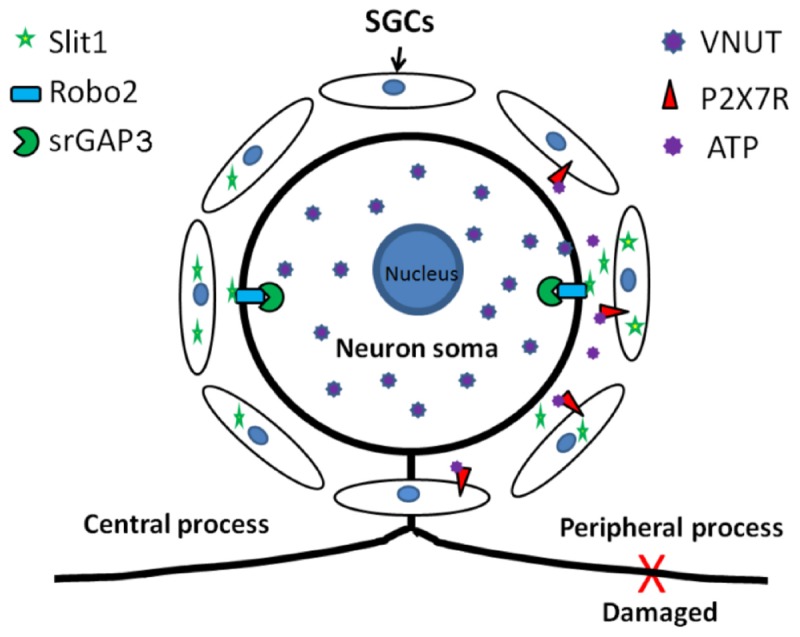
Schematic diagram of the role of Slit1 in the communication between neurons and SGCs. After peripheral nerve injury, neurons activate perineuronal SGCs, leading to upregulation of Slit1 expression through the VNUT-ATP-P2X7R pathway, and SGCs then promote the regeneration of injured neurons through the Slit1-Robo2-srGAP3 pathway (Zhang et al., 2010; Chen et al., 2012b).

Our experiments have demonstrated that an injury signal from DRG neurons can activate P2X7R on the SGC membrane and upregulate the expression of Slit1 in SGCs. These results show that the ATP-P2X7 pathway is involved in signal transduction from peripheral nerve injury to SGCs, leading to upregulation of Slit1 expression. Combined with our previous research (Zhang et al., 2010; Chen et al., 2012b), these findings indicate that Slit1 expressed in perineuronal SGCs can bind to Robo2 receptors on neuronal membranes and mediate neuronal process outgrowth through the Robo2-srGAP3 pathway. Our study complements the molecular mechanism of bidirectional signal transmission between neurons and SGCs in the anatomical unit of DRG neurons and clarifies the role of SGCs in nerve injury and regeneration, as shown in the schematic diagram (Figure 8).

## Materials and Methods

### Animals

Adult Sprague-Dawley (SD) rats of 250–300 g were purchased from the Experimental Animal Department of Hunan Agricultural University (Changsha, Hunan, China) and housed in a standard rat cage with unlimited access to water and food under 12 h of continuous light every 24 h. Animal scarification and tissue collection were approved by the animal ethics committee of Hainan Medical University (Haikou, China) and were performed according to the guidelines of the Chinese Care and Use legislation. All efforts were made to minimize animal suffering and the number of animals used.

### Surgeries, FG Injection, Brilliant Blue G (BBG) Intraperitoneal Injection, Tissue Preparation and Sectioning

SD rats were used to generate the SNC model as previously described (Zou et al., 2016). The rats were anesthetized using 2% sodium pentobarbital (40 mg/kg, intraperitoneal). The crush lesion was induced by non-serrated forceps with a smoothed surface. The forceps were instrumented with strain gauges and calibrated by a force-sensing resistor (FSR400, Interlink Electronics, CA, United States) linked to an Avometer. The applied force was 1000 g; the duration of the crush was 1 min; and the left sciatic nerve was subjected to a sham control surgery without crush injury. The wounds were sutured after surgery with nylon 5-0 sutures.

Three SD rats were used for retrograde tracing of Fluor-Gold (FG; Fluorochrome; 80014; Sigma-Aldrich, St. Louis, MO) as previously described (Li et al., 2016). The modified procedure was as follows: A glass micropipette (tip diameter 40–60 μm) connected to a microsyringe (1 μl, Hamilton, Reno, NV, United States) was inserted into the epineurium 2 mm from the distal point of the injured site of the sciatic nerve. A volume of 0.1 μl of 4% FG solution dissolved in normal saline was gradually pressure injected (continuously for over 1 min) into the target site. The needle tip was kept at the target site for another 10 min to avoid FG leakage. Three rats were kept alive for 7 days after FG injection.

The preparation and administration of BBG (P2X7R antagonist) followed the procedures used in a previous study (Ryu and McLarnon, 2008). To prepare a 10 mg/ml BBG solution, 0.3 g of BBG powder (Sigma-Aldrich, 27815-25-f) was dissolved in 30 ml of 0.9% saline. After SNC, the BBG solution (30 mg/kg) was used for intraperitoneal injection at a dose of 50 mg/kg, and the animals that survived for 7 days were subjected to daily injection of this dose for 7 days. Control animals were injected with an equivalent amount of saline.

Following the operation, 30 rats were divided into five groups (*n* = 6) and kept alive for 1, 3, 7, 14 or 28 days. These rats were used for Slit1 western blotting (*n* = 3) and immunohistochemistry (*n* = 3) experiments. Six rats were used for VNUT and Slit1 western blotting at 7 days post-SNC with BBG injection. The remaining rats that were not used for western blotting were anesthetized with an overdose of sodium pentobarbital solution (100 mg/kg, i.p.) and perfused through the ascending aorta with 100 ml of 0.9% (w/v) saline, followed by 500 ml of 4% (w/v) paraformaldehyde and 30% (v/v) saturated picric acid in 0.1 M phosphate buffer (PB, pH 7.4).

L5-L6 DRG from both sides were removed and incubated in 4% paraformaldehyde for 3 h. The tissues were then dehydrated with sucrose density gradient buffer. DRG were embedded with optimum cutting temperature medium (O.C.T. Compound 4583, Tissue-Tek, SAKURA, CA United States), subjected to cryosectioning and kept at 4°C. Sections of the DRG were cut at a 10 μm thickness on a freezing microtome (Shandon Cryotome E, Thermo Electron Corp., United States). The sections were mounted on gelatine-coated glass slides, air dried, and kept at 4°C in the freezer for later use.

### DRG Cell Culture

Dorsal Root Ganglia cell culture was performed as described previously (Castillo et al., 2011). Following the administration of anesthesia through intraperitoneal injection with 2% pentobarbital solution (40 mg/kg), L5-L6 DRG were isolated from 3- to 4-week-old SD rats of both sexes. To minimize the interference caused by SCs, nerve roots at both ends of the ganglion were removed as much as possible. Additionally, to avoid interference from fibroblasts, the outer membrane of the ganglion should be removed completely. DRG neurons and glial cells were cultured in Dulbecco’s modified Eagle’s medium (Sigma, St. Louis, MO, United States) supplemented with 10% FBS (Sigma, St. Louis, MO, United States). After 4 h of culturing, cultured cells were fixed with 4% paraformaldehyde.

### Western Blotting

Rat dorsal root ganglia (DRG) (L5-L6) were homogenized with homogenization buffer (20 mM Tris, pH 8, 137 mM NaCl, 1% NP-40, 1 mM sodium orthovanadate, and protease inhibitor cocktail). The supernatant was removed and subjected to protein quantification with a Pierce BCA reagent kit (Thermo Fisher Scientific, 23227). Then, 50 μg of the protein lysate was loaded onto a 10% SDS-PAGE gel for electrophoresis and subsequently transferred to a polyvinylidene-fluoride (PVDF) membrane. After 4 h of blocking with a 5% milk-PBS solution, the membrane was incubated with the primary antibody at 4°C overnight. Thereafter, the membrane was washed three times and incubated with an HRP-conjugated secondary antibody. Membranes developed by incubation with β-actin, GAPDH and rabbit HRP-conjugated secondary antibodies (Table 1) were used as controls. Protein was visualized using the Pierce ECL reagent kit (Thermo Fisher Scientific, 32132). Quantitative analysis of proteins was carried out on the protein bands with ImageJ (National Institutes of Health) and Microsoft Excel (Microsoft Corp.). The amount of the Slit1 protein was normalized to the amount of the β-actin protein. The amount of the VNUT protein was normalized to the amount of the GAPDH protein. All the western blot experiments were repeated three times.

**TABLE 1:** Primary and secondary antibodies used in this study.

**Category**	**Antibody**	**Manufacturer**	**Catalog number**	**Dilution**
**Primary antibody**	Polyclonal mouse anti-Slit1	Abcam	Ab115892	1:200 IF 1:1000 WB
	Polyclonal rabbit anti-GS	Abcam	ab16802	1:400 IF
	Chicken polyclonal anti-MAP2	Abcam	ab5392	1:2000 IF
	Rabbit anti-ATF3	Santa Cruz	sc-188	1:800 IF
	Mouse monoclonal anti-ATF3	Abcam	Ab58668	1:100 IF
	Goat polyclonal anti-P2RX7	Abcam	Ab93354	1:100 IF
	Guinea pig polyclonal anti-VNUT	Millipore	ABN83	1:1000 IF 1:500 WB
	Rabbit polyclonal anti-ß-actin-loading control	Abcam	Ab1801	1:2000 WB
	Rabbit polyclonal anti-GAPDH-loading control	Abcam	Ab9485	1:2500 WB
**Secondary antibody**	Alexa Fluor 594-donkey anti-mouse IgG	Jackson	715-585-1	1:200 IF
	FITC-rabbit anti-chicken	Millipore	AP162F	1:100 IF
	Alexa Fluor 488-donkey anti-rabbit IgG	Jackson	711-546-1	1:200 IF
	Cy3-donkey polyclonal Anti Chicken IgY	Millipore	703-175-155	1:200 IF
	Alexa Fluor 488-donkey anti-goat IgG(H + L)	Jackson	705-545-147	1:200 IF
	Alexa Fluor 594-donkey anti-rabbit IgG(H + L)	Jackson	711-585-152	1:200 IF
	Alexa Fluor 488-donkey anti-guinea pig IgG(H + L)	Jackson	706-545-148	1:200 IF
	Goat anti-mouse IgG(H + L) (HRP)	Abcam	Ab205719	1:10000 WB
	HRP-conjugated AffiniPure goat anti-guinea pig IgG(H + L)	Proteintech	SA00001-12	1:5000 WB
	Goat anti-rabbit IgG(H + L) (HRP)	Abcam	Ab205718	1:10000 WB

Primary and secondary antibodies used for immunohistochemistry, immunocytochemistry and western blotting in this study (Table 1).

### Immunohistochemistry

Dorsal Root Ganglia sections were incubated with 5% donkey serum for 1 h at room temperature before being incubated with the primary antibodies in the refrigerator overnight. Subsequently, in a dark chamber, the sections were incubated with the secondary antibodies for 2 h at room temperature. Sections incubated with 2% donkey serum without a primary antibody were used as a negative control.

Slit1, MAP2, GS, ATF3, VNUT and P2X7 was used for single labeling of DRG sections, and Slit1 was used for single labeling of sections of 7-day DRG subjected to retrograde tracing with FG. VNUT was used for single labeling in sections of DRG at 7 days post-SNC, and dual labeling of GS and Slit1 was performed in sections from the intact DRG and injured DRG. MAP2 and Slit1 dual labeling was performed in sections from intact DRG. Dual labeling with ATF3 (Santa Cruz, sc-188) and Slit1 or ATF3 (Abcam, ab58668) and VNUT was performed in sections of DRG at 7 days post-SNC. Dual labeling of GS and P2X7 or GS and Slit1 was performed sections of DRG at 7 days post-SNC with BBG injection.

### Immunocytochemistry

Staining for immunocytochemistry was carried out as described previously (Castillo et al., 2011). In brief, slides with fixed cells were blocked with 5% donkey serum and permeabilized in 0.1% Triton-X-100 PBS buffer. Then, the slides were incubated with primary antibodies at 4°C overnight and subsequently incubated with secondary antibodies for 2 h at room temperature. DAPI (4’, 6-diamidino-2-phenylindole, Boster, AR1176, China) was used to stain nuclei. Slides incubated with 2% donkey serum without a primary antibody were used as a negative control.

GS and Slit1, MAP2 and Slit1 were dual labeled in the cultured DRG cells.

### Semi-Quantitative Analysis of the Immunofluorescence Images

All the sections were first used for quantitative analysis and determination of the relative changes in the immunofluorescence intensity in each group. The same slide was used to avoid bias regarding slide-to-slide variations in labeling intensity. Similar trends in Slit1 expression were observed between the five experimental groups for all animals in each experimental group. The fluorescence images of DRG sections (Slit1 and GS double-labeled) subjected to BBG treatment were captured by laser scanning confocal microscopy (FV1000, Olympus, Japan), and fluorescence images of the other groups were captured with a fluorescence microscope (Olympus IX51 and Olympus BX51, Japan) by photographing each DRG slide under the same conditions with the same exposure. Semi-quantification of the fluorescence intensity was performed on images captured with 20× or 40× objective lenses using CellSens Dimension software (Olympus, Japan) integrated with the fluorescence microscope.

The method for the quantification of the fluorescence signal referred to a previous publication (Nadeau et al., 2014). For the profiling of Slit1 expression levels in SGCs, the processing and analysis of fluorescence signals from the cytoplasm of the SGCs were performed as follows: fifteen L5 DRG from different time points and three DRG (randomly chosen from the contralateral side of the experimental condition) were analyzed to determine the Slit1 relative fluorescence intensity (RFI) in SGCs. Using ImageJ (National Institutes of Health), fluorescence images with similar numbers of neurons obtained with a 20-fold objective lens were converted into 8-bit gray-scale images, and the background was subtracted (50 pixels selected). Neurons were individually selected with the “Free Selection Tool” in Photoshop software (Adobe Systems Inc.), and the images were deleted and saved, then inverted with ImageJ. The relative fluorescence intensity of the processed images was analyzed with ImageJ software. The obtained data are expressed as the mean and standard error of the mean (SEM). The data of each group were normalized to the data of the corresponding control group, and the obtained data were mapped by Excel software (Microsoft Corp.).

For the profiling of Slit1 protein expression levels in SGCs surrounding FG-positive and FG-negative neurons, a total of 1453 neurons from L5 to L7 days post-SNC DRG (*n* = 3) were used. The immunofluorescence signals in the cytoplasm of the SGCs surrounding the 688 FG-positive and 765 FG-negative neurons were traced separately, with the obtained values representing the average SGC labeling intensity around each respective neuron. The semi-quantified Slit1 protein levels in SGCs surrounding 723 AFT3-positive and 751 AFT3-negative neurons and the levels of VNUT protein on 955 AFT3-positive neurons and 896 AFT3-negative neurons were analyzed using the same method. The obtained data were expressed as the mean ± SEM.

For confocal fluorescence images of cultured cells, 100 40-fold objective fluorescence images were taken for analysis. SGCs contacted with neuronal soma were grouped into population 1 and non-contacted into population 2. A total of 347 SGCs of population 1 and 702 SGCs of population 2 were used to measure the average RFI of Slit1 in each satellite cell. The Slit1 RFI was expressed as the mean ± SEM. Slit1 RFI of population 1 SGCs was normalized by population 2 SGCs.

The processing of images and data analysis were performed with Photoshop CS5.1 (Adobe Systems Inc.), ImageJ (National Institutes of Health) and Microsoft Excel (Microsoft Corp.).

### Neuron Counting

For Slit1, similar qualitative trends were observed between the five experimental groups for all animals in each experimental group (*n* = 3). Three slides from five different groups of animals were selected for the experimental analysis of Slit1 such that similar numbers of neurons were present in the DRG sections representing each of the five experimental groups (*n* = 3). The numbers of neurons were counted in the fluorescence images of DRG slices from different time points, and the image pixels of SGCs around the intact neuronal soma were defined as the background. Neurons with a fluorescence intensity three times stronger than the background were defined as strongly positive neurons, and their numbers were counted with CellSens Dimension software. The number of neurons is expressed as the mean ± SEM.

### Statistical Analysis

Statistical analysis was performed with SPSS18.0 software (Statistical Product and Service Solutions 18.0, Al Monk, NY, United States). Paired *t* tests were used for comparisons between paired data. All the other data were analyzed using one-way repeated measures ANOVA. *P*-values of less than 0.05 were considered statistically significant.

## Data Availability

The datasets generated for this study are available on request to the corresponding author.

## Ethics Statement

Animal Subjects: The animal study was reviewed and approved by Animal scarification and tissue collection were approved by the animal ethics committee of Hainan Medical University (Haikou, China) and were performed according to the guidelines of the Chinese Care and Use legislation.

## Author Contributions

QP Z and XN Y designed the experiment, carried out data analysis, and wrote the manuscript. QP Z, JH Z, J Sh, XF Z, R R, ZJ M, YB H, Q K, YS W, X D, J Su, and ZZ L performed the experiment. XN Y provided the experimental reagents and access to the data analysis software.

## Conflict of Interest Statement

The authors declare that the research was conducted in the absence of any commercial or financial relationships that could be construed as a potential conflict of interest.
