# Supplementary material for: The ATP-P2X7 Signaling Pathway Participates in the Regulation of Slit1 Expression in Satellite Glial Cells
Source: Front Cell Neurosci. 2019 Sep 19;13:420. doi: 10.3389/fncel.2019.00420 (PMC6761959; doi:10.3389/fncel.2019.00420)
Supplement: Supplementary file 1 [file Table_1.DOCX]

Supplementary Material

## Supplementary Figures


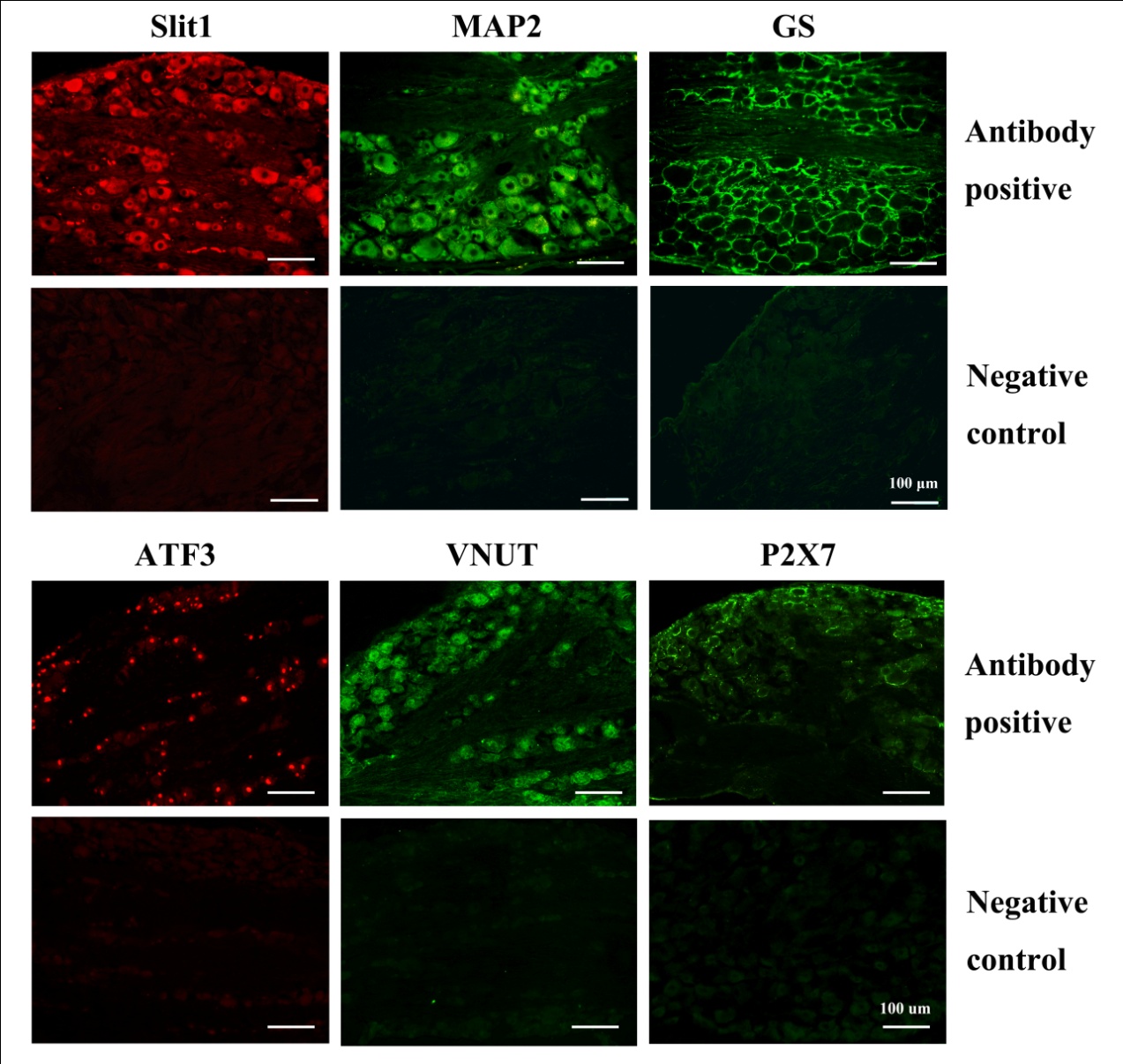


**Supplementary Figure 1.** Confirmation of antibody specificity. Line 1 images show positivity for the Slit1 (red), MAP2 (green) and GS (green) antibodies in DRG sections by immunofluorescence staining; line 2 images are for the corresponding negative controls. Line 3 images show positivity for the ATF3 (red); VNUT (green) and P2RX7 (green) antibodies in DRG sections by immunofluorescence staining; line 4 images show the corresponding negative controls. Scale bar = 100 µm.


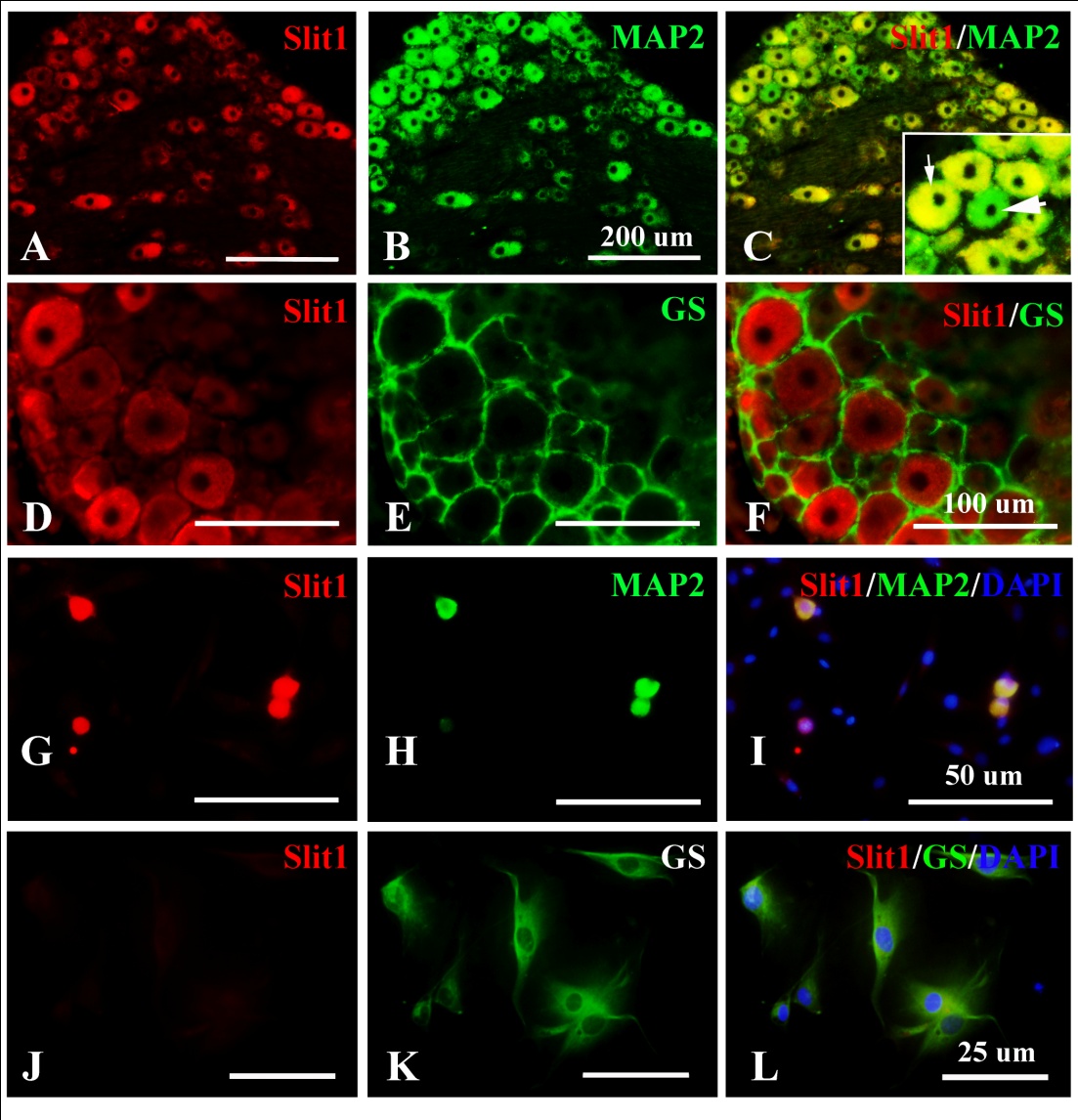


**Supplementary Figure** **2**. Expression of Slit1 in DRG, cultured primary sensory neurons and glial cells was demonstrated by double immunofluorescence labelling.**(A-C)** DRG sections, Slit1 (red) and MAP2 (green) immunofluorescence staining; C shows a merged image of images A and B; the small arrow indicates neurons with co-expression of MAP2 and Slit1. Scale bar = 200 µm. **(D-F)** DRG sections, Slit1 (red) and GS (green) immunofluorescence staining; F shows a merged image of images D and E. Scale bar = 100 µm. **(G-I)** Immunofluorescence staining of Slit1 (red) and MAP2 (green) in cultured primary DRG neurons; (I) Merged image of Slit1, MAP2 and DAPI immunofluorescence images; DAPI labels the nucleus; scale bar = 50 um; **(J-L)** Immunofluorescence staining of Slit1 (red) and GS (green) in cultured DRG SGCs; (L) Merged image of Slit1, GS and DAPI immunofluorescence images; DAPI labels the nucleus; scale bar = 25 µm.


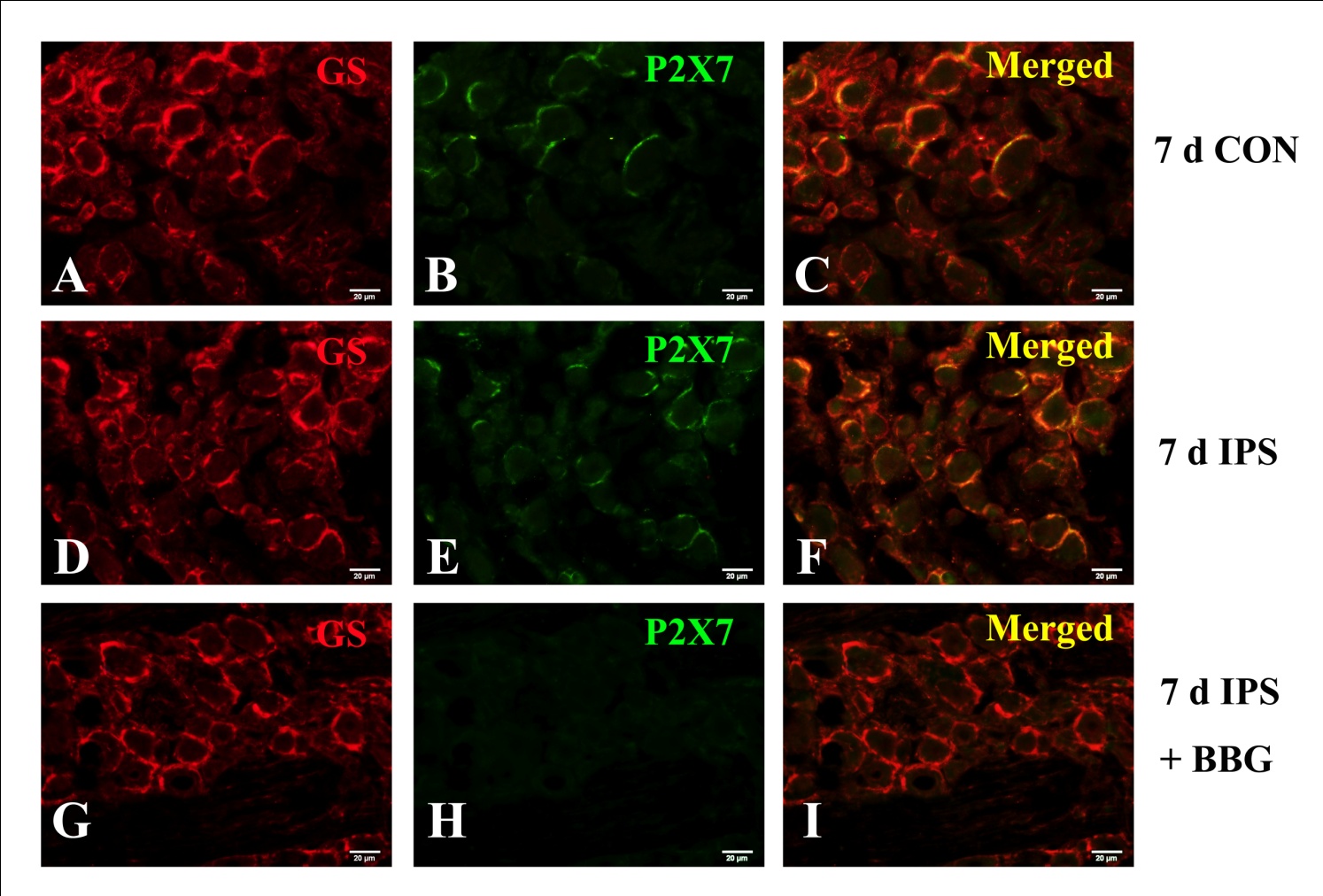


**Supplementary Figure 3.** Double immunofluorescence staining of GS and P2X7R in day 7 DRG. **(A-C)** Contralateral DRG sections from the day 7 group. **(D-F)** Ipsilateral DRG sections of the day 7 group. (**G-I)** Ipsilateral DRG sections of the day 7 group subjected to peritoneal injection of BBG. (A, D, G) GS (red) immunofluorescence staining. (B, E, H) P2X7R (green) immunofluorescence staining. (C, F, I) Merged images of image A and image B, image D and image E, and image G and image H. Scale bar = 20 µm.


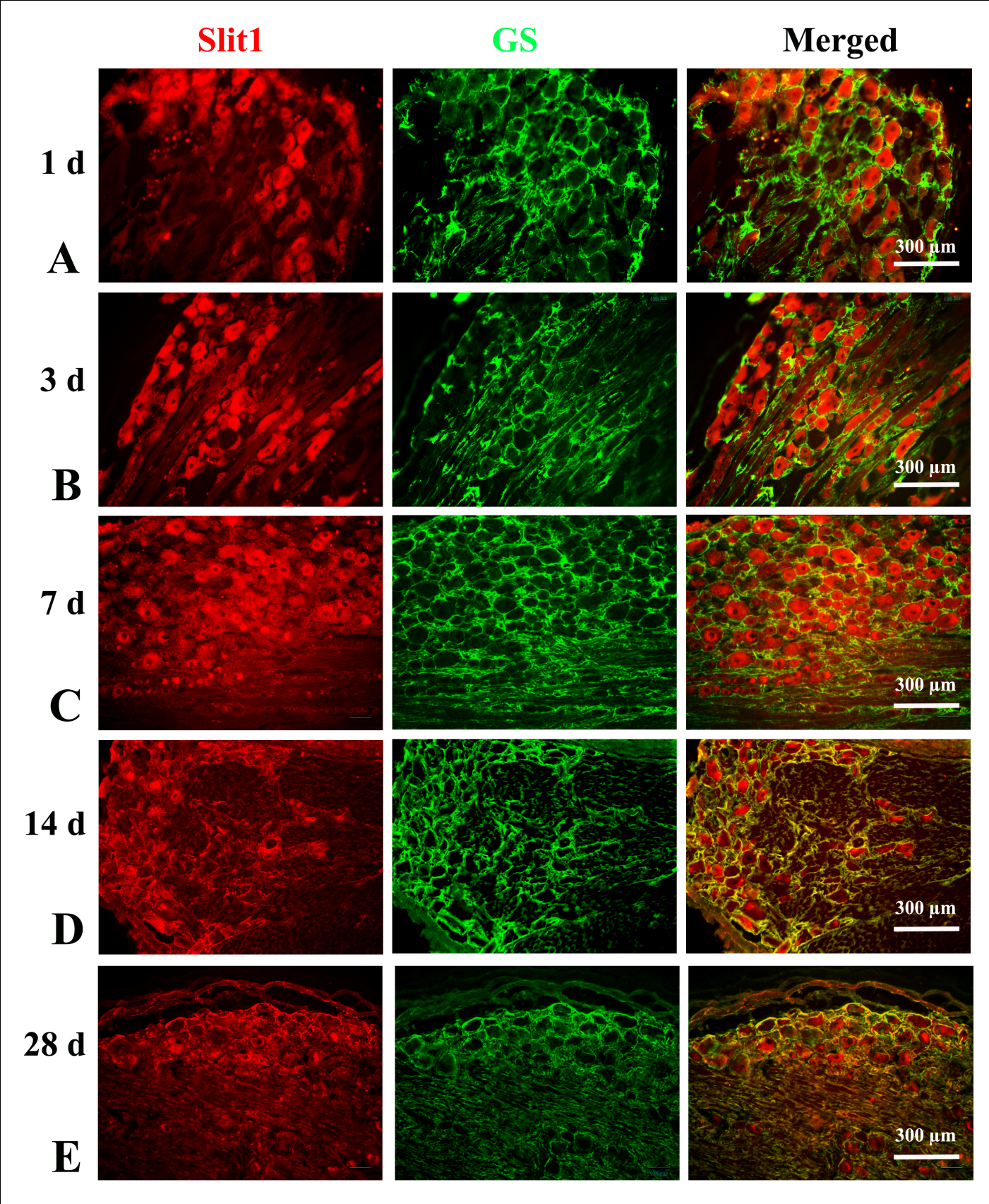


**Supplementary Figure 4.** Slit1 expression in SGCs of injured DRG was detected by Slit1 and GS immunofluorescence double staining. Line A, B, C, D and E were double-labeled immunofluorescence images of the injured DRG on 1d, 3d, 7d, 14d and 28d, respectively. The left column is Slit1 fluorescent image (red), the middle column is GS fluorescent image (green), and the right column is fusion image. Scale bar = 300 µm.

**
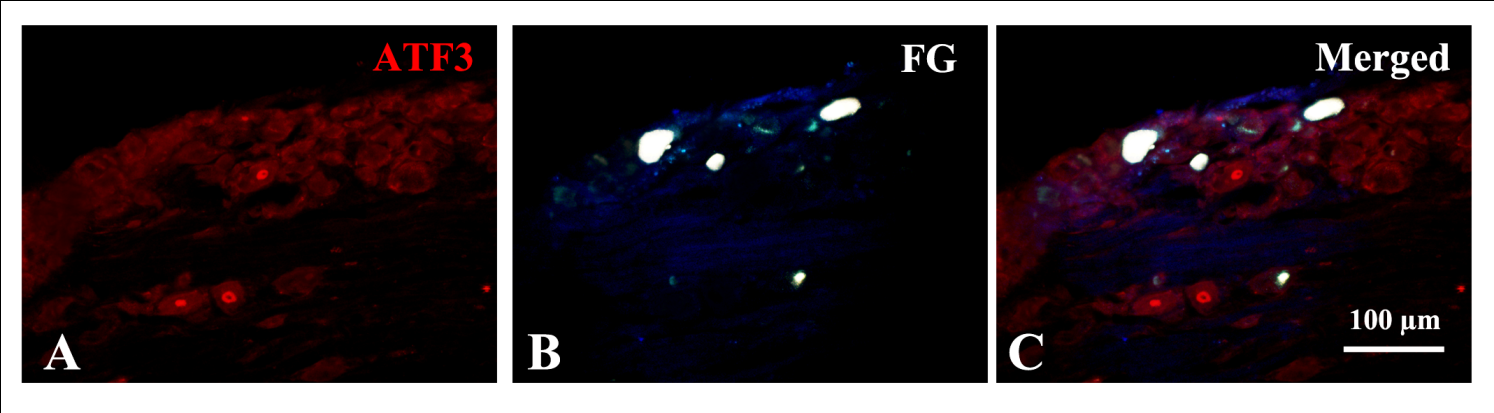
**

**Supplementary Figure 5.** ATF3 immunofluorescence assay showed that ATF3-positive neurons did not superimpose with FG-labeled neurons in injured DRG after retrograde tracing of FG. **(A)** ATF3 fluorescent image (red). **(B)** FG tracer fluorescence image (white). **(C)** Merged image of picture A and B. Scale bar = 100 µm.

## Supplementary Table 1

The analytical method proposed in this paper was applied to the expression frequencies of 3 markers (Slit1, VNTUT, and ATF3) in adult rat DRG neurons of 7 d post-SNC. Column 1 shows the input data used for DRG neurons was come from the fluorescence double-label staining data of Slit1 and ATF3, VNUT and ATF3 in this study. Columns 3 report the frequency ranges recovered for DRG neurons, using original data from this study.

| (1) Expression frequency of one and two markers | (2) Molecular profile | (3) DRG neurons recovered frequency ranges |
| --- | --- | --- |
| P_Slit1_ = 0.1.5 ± 0.026 | Slit1^-^VNUT^-^ATF3^-^ | 0.366 – 0.568 |
| P_VNUT_ = 0.292 ± 0.02 | Slit1^+^VNUT^+^ATF3^+^ | 0 - 0.131 |
| P_ATF3_ = 0.512 ± 0.08 | Slit1^-^VNUT^+^ATF3^+^ | 0.141 – 0.312 |
| P_Slit1/ATF3_ = 0.158 ± 0.029 | Slit1^-^VNUT^-^ATF3^+^ | 0 – 0.32 |
| P_VNUT/ATF3_ = 0.292 ± 0.02 | Slit1^-^VNUT^+^ATF3^-^ | 0 – 0.04 |
|  | Slit1^+^VNUT^-^ATF3^+^ | 0 – 0.131 |

The results obtained from the Supplementary Table 2 (software provided by Luigi Catacuzzeno) showed that subpopulations Slit1^+^VNUT^-^ATF3^-^ and Slit1^+^VNUT^+^ATF3^-^ do not exist, and probably also subpopulation Slit1^-^VNUT^+^ATF3^-^ does not exist, since its frequency is still very small. The results also showed that subpopulations Slit1^-^VNUT^-^ATF3^-^ and Slit1^-^VNUT^+^ATF3^+^ are the most representative of DRG neurons of 7 d post-SNC and are the only one the data may demonstrate the existence (their lower bound is not zero).
